# Effect of intravenous thrombolysis on core growth rate in patients with acute cerebral infarction

**DOI:** 10.3389/fneur.2023.1096605

**Published:** 2023-02-23

**Authors:** Xueqi Wang, Hao Zhang, Qi Wang, Gang Li, Hao Shen, Yaping Xiao, Luran Xu, Yuming Long, Chen Chen, Zhengyu Huang, Yue Zhang

**Affiliations:** Shanghai East Hospital, Tongji University, Shanghai, China

**Keywords:** acute ischemic stroke, intravenous thrombolysis, core growth rate, collateral circulation, large vessel occlusion, reperfusion, therapy

## Abstract

**Objective:**

This study aimed to investigate the effects of recombinant tissue plasminogen activator intravenous thrombolysis (IVT) on the core growth rate of acute ischemic stroke.

**Methods:**

Stroke patients with large vessel occlusion and non-recanalization from IVT treatment were retrospectively included in this study and divided into two groups: IVT and non-IVT. The core growth rate was estimated by the acute core volume on perfusion CT divided by the last known well time from stroke to CT perfusion. The primary endpoint was the core growth rate, the tissue outcome was 24 h-ASPECTS, and the clinical outcome was a 3-month modified Rankin score.

**Results:**

A total of 94 patients were included with 53 in the IVT group and 41 in the non-IVT group. There was no significant difference in age, gender, hypertension, diabetes, atrial fibrillation, acute NIHSS, and last known well time from stroke to CT perfusion acquisition between the two groups. The core growth rate in the IVT group was lower than that in the non-IVT group, which was statistically significant after multivariate adjustment (coefficient: −5.20, 95% CI= [−9.85, −0.56], *p* = 0.028). There was a significant interaction between the IVT and the collateral index in predicting the core growth rate. The analysis was then stratified according to the collateral index, and the results suggested that IVT reduced the core growth rate more significantly after the worsening of collateral circulation (coefficient: 15.38, 95% CI= [−26.25, −4.40], *p* = 0.007). The 3-month modified Rankin score and 24 h-ASPECTS were not statistically significant between the two groups.

**Conclusion:**

Intravenous thrombolysis reduces the core growth rate in patients with AIS, especially those with poor collateral status.

## 1. Introduction

Intravenous thrombolysis (IVT) is an established treatment for acute ischemic stroke (AIS), and it can be rapidly initiated after clinical assessment and cranial CT scan (1–3). However, IVT also has significant limitations, such as the patients' need to receive IV tPA within 4.5 h of the onset, and the recanalization rates are low in patients with large vascular occlusion (LVO), that is, a meta-analysis reported approximately 35% for M1 MCA occlusions, 13% for ICA occlusions, and 13% for BA occlusions (4).

Since 2015, several clinical trials acknowledged the superiority of endovascular thrombectomy (EVT), which has a higher rate of recanalization of LVO and a longer treatment window (5–9). Therefore, the “bridge” therapy was proposed to use EVT to rescue patients with a lack of recanalization after IVT. Some clinical trials showed that early administration of alteplase can promote microvessel patency and that the rate of successful recanalization in bridge therapy was significantly higher than that in patients who only accept EVT (10, 11). However, IVT may increase the risk of bleeding (12) and promote thromboplastic migration (13). Hence, whether patients with LVO-AIS who arrive at the hospital within 4.5 h of the onset can benefit from the “bridge” therapy is a research hotspot (14, 15).

In patients with AIS, successful recanalization and core volume are the strongest predictors of outcome (16). Before vessel recanalization, the infarct core increases linearly within 6 h of stroke onset (17, 18). Assuming that the core at the time of stroke onset was 0, the infarction volume divided by the time from stroke onset to CTP can be used to estimate the speed of cerebral infarction progression. The core growth rate has been reported to be an independent predictor of clinical outcomes and is highly associated with the collateral status. Reducing the core growth rate may reduce the volume of core infarction and may have important therapeutic implications on AIS.

This study aimed to assess the efficacy of IVT on changing infarct core growth rate in patients with LVO-AIS who had not achieved recanalization.

## 2. Materials and methods

### 2.1. General information

We conducted a retrospective cohort study that involved 94 AIS patients with LVO between January 2017 and March 2021 at the Shanghai East Hospital—Department of Neurology. All the patients were divided into two groups according to whether or not they received IVT.

### 2.2. Inclusion criteria

Patients were selected based on the following criteria: (1) All the patients met the diagnostic criteria of acute cerebral infarction due to LVO; according to the guidelines, the IVT group patients met the indications of IVT, and the contraindications of IVT were excluded; (2) the last known well time from stroke to completion of CTP was <6 h; (3) the ischemic core volume on the CTP was <70 ml, penumbra ≥10 ml, and radio > 1.2; and (4) the clinical information was complete.

### 2.3. Methods

The clinical data of patients included age, gender, and histories of hypertension, diabetes, heart issues, prior stroke, current smoking, and NIHSS recorded at the hospital arrival, as well as radiographic data. The DT collateral index was used to evaluate the collateral status (19).

The IVT group patients were given 0.9 mg/kg of rt-PA for thrombolysis: 10% of the total dose was given intravenously for 1 min, while the remaining dose was injected intravenously 1 h later.

### 2.4. Imaging acquisition and post-processing

Baseline CT imaging included brain non-contrast CT, CTP, and CTA, obtained with different CT scanners (64, 128, 256, or 320 detectors, with Toshiba [Tokyo, Japan], Siemens [Munich, Germany], or GE [Cleveland, OH, USA] scanners). The axial coverage ranged from 80 to 160 mm.

The CTP data were processed by commercial software MIStar (Apollo Medical Imaging Technology, Melbourne, Vic, Australia). CTP parameters were generated by applying the mathematical algorithm of singular value decomposition with delay and dispersion correction (20, 21). The following four CTP parameters were generated: cerebral blood flow (CBF), cerebral blood volume (CBV), mean transit time (MTT), and delay time (DT). The penumbra and core volume were measured on acute CTP with dual threshold setting (22): DT at the threshold of 3 s for whole ischemic lesion volume and CBF at the threshold setting of 30% for acute core volume. The collateral index was defined by the ratio of DT >6 s/DT >2 s volume.

### 2.5. Calculation of the ischemic core growth rate

The core growth rate was determined using the baseline core volume divided by the time between the symptom onset and the CTP. It was assumed that the core volume was zero just prior to symptom onset and would grow in a near-linear pattern within 6 h of stroke onset (23). This study calculated the core growth rate using the following approach:

Core growth rate = Acute core volume on CTP / Last known well time from stroke to CTP (19, 24).

### 2.6. Statistical analysis

Data were statistically analyzed using the statistical software SPSS 25.0. The normality of the continuous variables was examined using the Kolmogorov–Smirnov test. When the normality assumption was not met, a non-parametric test was used. The categorical variables were compared by the chi-square test. Multi-factor linear regression was used to analyze predictors of ischemic core growth rate. Variables with a *p* < 0.25 were included in the regression model. In addition, collateral circulation was included in the model as a significant predictor. A scatter diagram was used to describe the interaction between IVT and DT collateral index in predicting the core growth rate. Then, the core growth rate of IVT vs. non-IVT patients was plotted across the collateral index (with a 0.100 increment). Within each collateral index category, the predictive power of IVT (vs. non-IVT) on the patient core growth rate was assessed by regression models.

### 2.7. Tissue outcomes

Alberta stroke program early CT score (ASPECTS) was used to evaluate the extent of anterior circulation infarction (25). The posterior circulation stroke was assessed by pc-ASPECTS (26). A normal CT scan has an ASPECTS value of 10 points. ΔASPECTS is defined as 24 h-ASPECTS minus baseline ASPECTS, which would be used to describe the change in brain tissue. Multi-factor logistic and linear regression was used to describe the relationship between intravenous therapy and clinical outcomes and tissue outcomes.

### 2.8. Patient outcomes

The primary endpoint was the core growth rate. The clinical outcome was the modified Rankin score (mRS) at three months. The good clinical outcome was defined by an mRS score of 0–2 vs. an mRS score of 3–6, and the poor clinical outcome was an mRS score of 5–6 vs. an mRS score of 0–4.

## 3. Results

**Figure 1:**
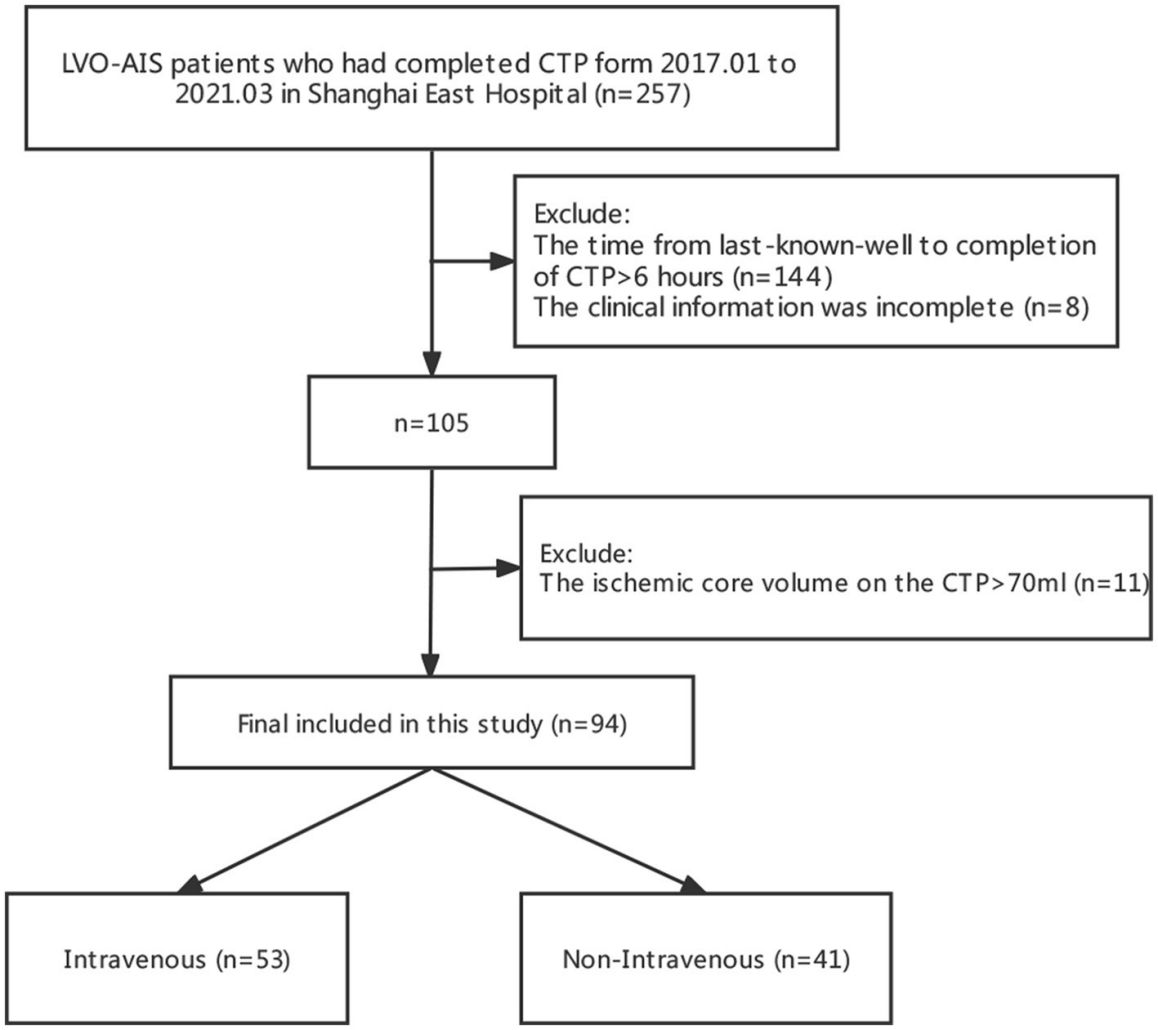
A flow diagram of the study.

The flow diagram of the study is given in Figure 1. A total of 94 patients met the inclusion criteria, of whom 53 patients received IVT and 41 patients were treated without IVT.

The baseline characteristics of the patients were similar in the two groups (Table 1). The average age of the patient was 39–98 years, and 57 patients (61%) were men. The median baseline NIHSS was 16 (IQR = 7). The median last known well time from stroke to CTP was 3.18 h (IQR, 2.27–3.98) in the IVT group and 3.4 h (IQR, 1.88–4.57) in the non-IVT group. There was no statistical difference between the two groups in age, gender, or AIS risk factors such as hypertension, diabetes mellitus, atrial fibrillation, previous history of stroke, or current smoking (P>0.05). Neither infarct volume nor collateral index showed a significant difference between the two groups (*p* = 0.982 and *p* = 0.760). No between-group differences were found at baseline ASPECTS or ΔASPECTS (*p* = 0.698 and *p* = 0.682). Although the difference was not statistically significant, 24 h-ASPECTS in the IVT group was slightly higher (7 vs. 6).

**Table 1:** Characteristics of the intravenous group vs. the non-intravenous group.

	**Intravenous (*n* = 53)**	**Non-intravenous (*n* = 41)**	**P-value**
Age, median (IQR)	71 (63–83)	71 (65–79)	0.565
Men, % (*N*)	58.5 (31/53)	63.4 (26/41)	0.628
Atrial fibrillation, % (*N*)	43.4 (23/53)	48.4 (20/41)	0.603
Hypertension, % (*N*)	64.2 (34/53)	46.3 (19/41)	0.084
Diabetes, % (*N*)	26.4 (14/53)	19.5 (8/41)	0.433
current smoking, % (*N*)	20.8 (11/53)	22.0 (9/41)	0.888
Prior Stroke, % (*N*)	22.6 (12/53)	34.1 (14/41)	0.216
Acute NIHSS score, median (IQR)	15 (12–19)	16 (12–19)	0.472
CBF <30, median (IQR)	14.0 (4.0–30.5)	18.0 (5.0–41.5)	0.235
Core growth, median (IQR)	4.52 (1.35–11.16)	5.91 (1.52–12.98)	0.430
Time from last-known-well to CTP acquisition (Hours), median (IQR)	2.83 (2.27–3.98)	3.98 (1.88–4.57)	0.064
DT > 6 s, median (IQR)	35 (16.5–61.0)	39 (13.5–55.5)	0.982
DT > 2 s, median (IQR)	176.0 (111.5–228.5)	203.0 (136.5–248.6)	0.595
Collateral index, median (IQR)	0.17 (0.12–0.32)	0.21 (0.12–0.31)	0.760
Baseline ASPECTS, median (IQR)	8 (8–9)	8 (7–9)	0.698
24 h-ASPECTS, median (IQR)	7 (6–8)	6 (5.75–7)	0.224
ΔASPECTS, median (IQR)	−1 (−2–0)	−1 (−2–0)	0.682
In-hospital mortality, % (*N*)	26.4% (14/53)	26.8% (11/41)	0.964
3-month modified Rankin score, median (IQR)	5 (1–6)	5 (2–6)	0.521
Good outcome rate, % (*N*)	34.0% (18/53)	26.8% (11/41)	0.314
Poor outcome rate, % (*N*)	50.9% (27/53)	56.1% (23/41)	0.581

IVT, intravenous thrombolysis; NIHSS, Scores on the National Institutes of Health Stroke Scale; IQR, interquartile range; CBF, cerebral blood flow; DT, delay time; AIS, acute ischemic stroke. Good outcomes and poor outcomes are defined by 3-month modified Rankin scores of 0–2 and 5–6, respectively. ΔASPECTS as 24 h-ASPECTS minus baseline ASPECTS.

Delay time collateral index (coefficient: 30.65, 95% CI= [14.37, 46.93], p<0.001) and intravenous therapy (coefficient: −5.20, 95% CI = [−9.85, −0.56], *p* = 0.028) showed significant differences. Intravenous may decrease the core growth rate of 5 ml/h for patients with stroke (Table 2).

**Table 2:** Intravenous effect for core growth rate.

	**Adjusted p-value**	**Coefficient**
Intravenous	0.028	−5.20
DT collateral index	<0.001	30.65
hypertension	0.598	−1.24
Prior stroke	0.700	−0.99

**Figure 2:**
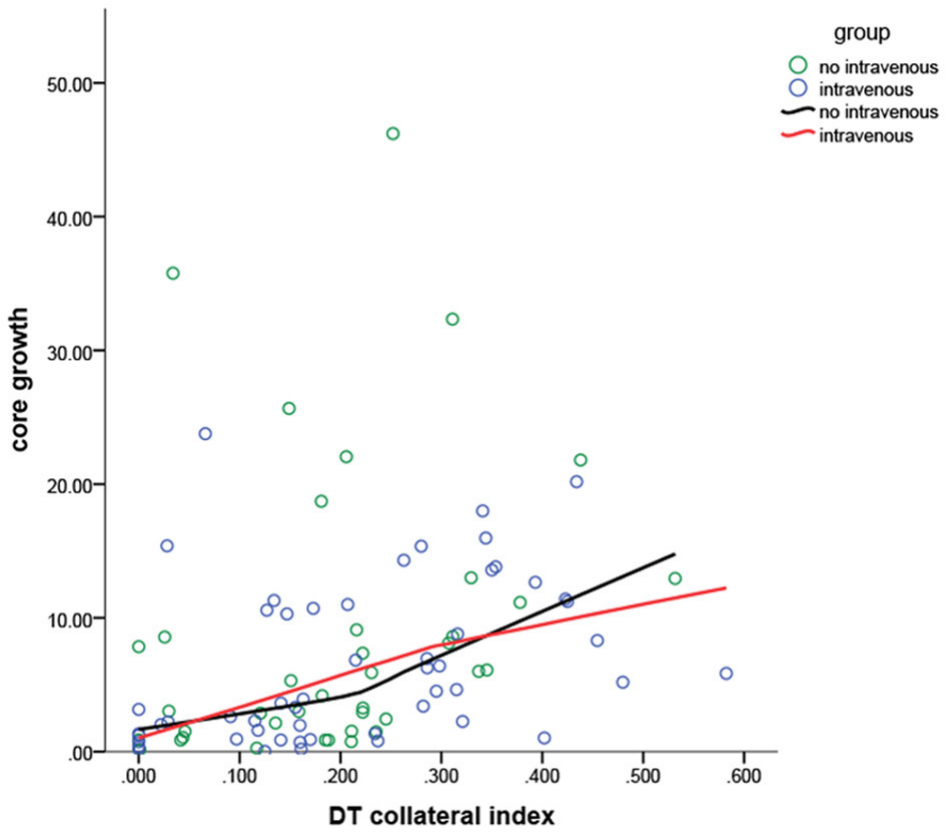
The core growth rate in different collateral indexes for the patients in the two groups. The IVT and non-IVT groups intersected two times, which suggested a significant interaction between the IVT and the collateral index, indicating that both of them interfere significantly with the core growth rate. The line of the non-IVT group showed a major increase when the DT collateral index was 0.250.

Figure 2 depicts the scatter plots for the association between the DT collateral index and the core growth rate in two groups.

**Figure 3:**
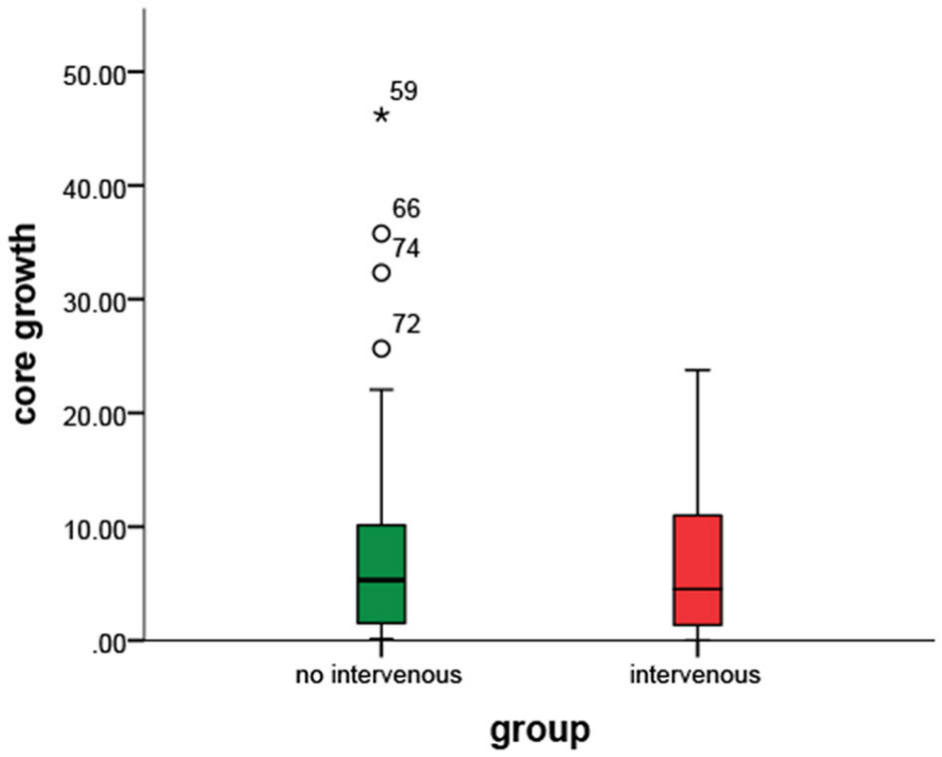
No clear difference was observed in the core growth rate between the two groups.

**Figure 4:**
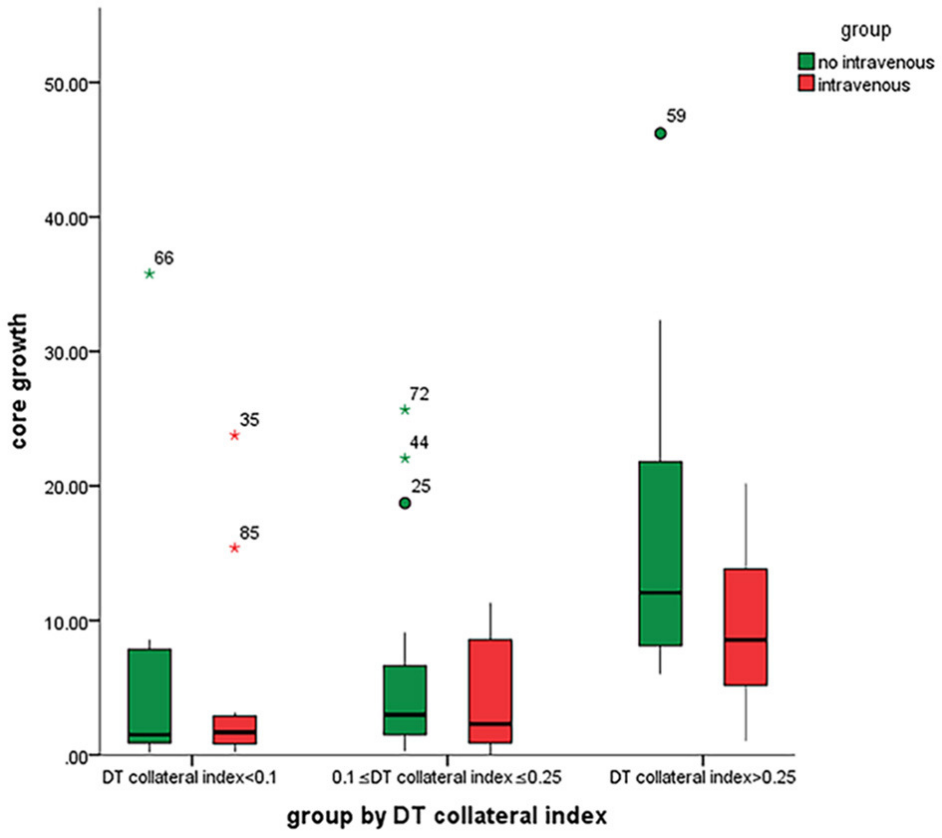
Differences in the core growth rate between within the IVT (or not) group and within a DT collateral index group. IVT significantly decreased the core growth rate in patients with poor collateral index.

Delay time collateral index was classified into three categories based on the core growth rate of the IVT vs. the non-IVT group (Figure 2). For DT collateral index <0.100 and 0.100–0.250, there was no statistical significance in the effect of IVT on the core growth rate (*p* = 0.616 and *p* = 0.426). For DT collateral index >0.25, after adjusting for DT collateral index, hypertension, and prior stroke, the IVT showed a statistically significant result on the core growth rate (coefficient: 15.38, 95% CI= [−26.25, −4.40], *p* = 0.007) (Table 3). In other words, for patients with poor collateral index, IVT may significantly decrease the core growth rate (Figures 3, 4).

**Table 3:** Predicting core growth rate by IVT across DT collateral index.

	**Adjusted p-value**	**Coefficient**
DT collateral index <0.1	0.616	−2.13
0.1 ≤ DT collateral index ≤ 0.25	0.426	−1.72
DT collateral index > 0.25	0.007	−15.38

Adjusted by DT collateral index, hypertension, and prior stroke.

Univariate and multivariate regression analyses were used to explore the association between intravenous therapy and clinical outcomes. There was no statistical difference in the 3-month modified Rankin score (Table 1). After adjusting for hypertension, prior stroke, and DT collateral index, both the good outcome (OR = 0.60, 95% CI = [0.205, 1.760]) and poor outcome (OR = 0.83, 95% CI = [0.355,1.953]) showed no significant predictive power between the two groups (Table 4). No significant between-group differences were detected in 3-month mortality (26.4 vs. 26.8%; OR=0.77, 95% CI = [0.314, 1.886]).

**Table 4:** Association between intravenous therapy and clinical outcomes.

	**P value**	**Adjusted p-value**	**Adjusted OR (95%CI)**
3-month good outcome	0.459	0.353	0.600 (0.205,1.760)
3-month poor outcome	0.620	0.673	0.832 (0.355,1.953)
3-month mortality	0.647	0.566	0.769 (0.314,1.886)
In-hospital mortality	0.979	0.603	0.768 (0.283,2.081)

Adjusted by hypertension, prior stroke, and DT collateral index.

After multivariate adjustments, the differences in 24 h-ASPECTS (coefficient: 0.451, 95% CI= [-0.281, 1.183], *p* = 0.224) and ΔASPECTS (coefficient: 0.183, 95% CI = [−0.501, 0.868], *p* = 0.595) were not statistically significant (Table 5).

**Table 5:** Association between intravenous therapy and tissue outcomes.

	**Adjusted p-value**	**Coefficient**
24 h-ASPECTS	0.224	0.451
ΔASPECTS	0.595	0.183

Adjusted by hypertension, prior stroke, and DT collateral index.

## 4. Discussion

In acute ischemic stroke (AIS) treatment, recanalization of the occluded vessel is crucial for a good clinical outcome (27). Intravenous thrombolysis (IVT) with rt-PA is a conventional treatment for AIS. However, the low vascular recanalization rate led to many conflicting views (4, 28). When clinical symptoms of patients with LVO-AIS did not significantly improve after receiving IVT, endovascular therapy was required to achieve recanalization. For such patients, the role of IVT remains controversial. This study showed that IVT may reduce the core growth rate in patients with AIS, even if the vessel did not achieve recanalization. Moreover, this effect was influenced by collateral circulation and was clearer in patients with poor collateral circulation.

Whether patients with AIS can benefit from the “bridge” therapy (BT) is a research hotspot. In a meta-analysis of 38 eligible observational studies, BT was associated with a higher likelihood of 3-month functional independence compared to direct mechanical thrombectomy (29). Whether IVT will extend the total time of recanalization therapy remains controversial. In recent years, numerous studies showed that the average recanalization time was not statistically different between the “bridge” therapy and direct thrombectomy (14, 15, 29), indicating that IVT did not delay the time to recanalization. In addition, with the application of tenecteplase, the time difference between the two treatment modalities was further reduced (30). This study demonstrated that IVT may reduce the core growth rate without vessel recanalization. Some studies also found that, in time-window patients who were transferred to an EVT capable center, the outcomes were better in patients who had previously received IVT (31). Therefore, IVT is considered the first-line treatment for patients with AIS even with LVO and IVT is required in those patients as early as possible.

Infarct core volume is an independent predictor for the outcome of patients with AIS. The smaller the infarct core, the better the likelihood of clinical outcomes (32). In recent years, several studies demonstrated that the infarct core grows linearly during the first 6 h of AIS (17, 18). Wheeler suggested that the early stroke core growth curves exhibited a nearly linear growth during the first 8 h after symptom onset for patients with <10% reperfusion (23). Therefore, the core growth rate in the first 6 h from the onset can predict the infarct core to some extent. In this study, all patients successfully completed the CTP examination in 6 h from last known well time. IVT was validated to reduce the infarct core growth rate in this study, and one possible mechanism is probably due to the reduction in thrombus volume by IVT (33, 34). In addition, the rt-PA can act on the distal microvasculature and reduce microvenous thrombosis (35). Thus, the blood supply in infarct areas can be improved. A retrospective study showed that the rate of successful recanalization was significantly higher in patients who received IVT before mechanical thrombectomy (11), and it might be implicated in those mechanisms. In this study, we found that IVT may reduce the infarct core growth rate, and patients with AIS who had a lower collateral circulation and underwent IVT exhibited slower infarct growth rates. The impact of collateral circulation on infarct core growth is well established (19). Better collateral circulation indicates slower infarct growth, while the effect of IVT is not perfect. However, in patients who had poor collateral circulation, along with a decrease in the thrombosis volume and thrombus load in the local microcirculation, the blood flow might have improved more in the ischemic penumbra.

There was no statistical difference in a 3-month modified Rankin score between the two groups. The potential explanation offered might be that, although the rate of core growth was decreased in the IVT group, the clinical outcome was largely decided by the developed infarct core volume and the degree of recanalization. Several studies confirmed that collateral circulation is the factor that has the greatest impact on the growth of infarct core (36, 37). Moreover, the factors associated with collateral circulation were age, smoking, hypertension (38), and the use of statins (39, 40). All these factors had no statistical significance in this study, which may have resulted in no difference in collateral circulation between the two groups. All patients in this trial had LVO, and they accepted mechanical thrombectomy after the CTP examination, and the intraoperative recanalization levels have been shown to impact the prognosis of stroke (41). Thus, the follow-up treatments might heavily influence the clinical outcome. It is difficult to assess the precise relationship between IVT with patient prognosis. Further studies may be needed to elucidate this relationship in a future study.

Although the 24 h-ASPECT in the IVT group was slightly higher than that of the non-IVT group, there was no significant difference in the tissue outcomes. Two considerations may have contributed to this result. On the one hand, despite this study finding that IVT may reduce the core growth rate in patients with LVO, the final infarct core was largely decided by the collateral circulation and the efficacy of endovascular therapy. On the other hand, the sample size in our study is relatively small, which needs to be expanded for a more in-depth research in future, and the effects of intravenous thrombolysis on histological changes would have likely been observed.

This retrospective single-center study has several limitations. First, the lack of randomized treatment allocation in this study was the main limitation. Second, there were no clear differences in baseline core growth rates between the two groups, which might be affected by the collateral circulation. We further adjusted it by the multivariate regression models and removed the effects of the collateral index, which showed positive results. Third, this study was conducted with a small sample size, which might have led to a certain degree of bias. Fourth, some of the patients had unwitnessed onset and the time of stroke onset was uncertain. Hence, the estimation of time from onset to CTP may be extended.

Although the difference in the long-term prognosis of the patients was not observed, the present study provided important new information about the benefit of IVT. “Time is brain” in this study, and IVT successfully reduced the core growth rate, suggesting the underlying application in the treatment of acute cerebral infarction. For patients with AIS who arrive at the hospital within the time window, we suggest IVT in the absence of contraindications and expect more benefits from the “bridge” therapy. Further studies are required to confirm these conclusions.

## Data availability statement

The raw data supporting the conclusions of this article will be made available by the authors, without undue reservation.

## Ethics statement

The studies involving human participants were reviewed and approved by Ethics Committee of the Shanghai East Hospital. The patients/participants provided their written informed consent to participate in this study.

## Author contributions

YZ and ZH contributed to the conception and design of the present study. XW and HZ contributed to drafting a significant portion of the manuscript or figures. QW, HS, YX, and LX contributed to the acquisition and analysis of data. YL, CC, and GL helped perform the analysis with constructive discussions. All authors contributed to the article and approved the submitted version.
